# Persistent Diarrhoea after Percutaneous Endoscopic Gastrostomy (PEG) in Paediatric Patient: Lessons from a Complication

**DOI:** 10.1155/2022/7663038

**Published:** 2022-06-10

**Authors:** Sara Silvaroli, Filomena Valentina Paradiso, Valentina Giorgio, Lorenzo Nanni

**Affiliations:** ^1^Unit of Paediatric Surgery, Department of Woman and Child Health and Public Health, Fondazione Policlinico Universitario A. Gemelli IRCCS, Largo Agostino Gemelli 8, Rome 00168, Italy; ^2^Unit of Paediatrics, Department of Woman and Child Health and Public Health, Fondazione Policlinico Universitario A. Gemelli IRCCS, Largo Agostino Gemelli 8, Rome 00168, Italy

## Abstract

Percutaneous endoscopic gastrostomy (PEG) is increasingly used in paediatric population. We report a case of a 4-year-old boy who, two weeks after PEG placement, presented persistent diarrhoea interpreted as intolerance to enteral feeding. His CT scan confirmed the correct placement of gastrostomy, but during gastroscopy, gastrostomy could not be found in the stomach, and the following colonoscopy revealed migration of gastrostomy to the transverse colon. The patient required removal of the misplaced PEG and conservative management of the fistula with surgical replacement of gastrostomy. We faced an unusual presentation of PEG placement complication due to colon interposition during blind gastric puncture. In children with anatomical deformities, previous surgery, or low weight or malnutrition (<10 kg), we suggest laparoscopic-assisted gastrostomy to avoid the risk of a major complication.

## 1. Introduction

Currently, gastrostomy in children is performed in a wide spectrum of situations, including feeding disorders in neurologically impaired children, nutrition and fluid supplementation in metabolic disorders, and enteral nutrition in short gut syndrome. Gastrostomy is generally indicated when poor oral intake is likely to persist for more than 3 months [1]. In all these patients, the nutritional status, at time of placement, is compromised, and the quality of healing is not always optimal [2].

Gastrostomy can be accomplished with various procedures, i.e., the Stamm open procedure, percutaneous endoscopic gastrostomy (PEG), or laparoscopic-assisted gastrostomy (LAG).

PEG has become an effective and well-accepted method for enteral feeding in children. PEG tube insertion became the preferred method because it is minimally invasive, fast to perform, and correlates to low costs and high patient tolerance. In addition, PEG can be performed with minimal anaesthetic exposure outside the operating room. This can be especially helpful for patients who might not tolerate deep anaesthesia. This procedure is generally safe, correlated to great parental satisfaction and predominantly minor complications [3]. However, rates of paediatric PEG complications vary widely in literature ranging from 4 to 44% [2]. Minor problems may include wound infection, granulation tissue, and leakage. Major complications may include peritonitis, haemorrhage, intestinal obstruction, buried bumper, and gastroenteric fistula formation. Major complications, which require reoperation, range from 3% to 5% [4], among them is reported the migration of the PEG tube into the transverse colon, which can remain unrecognized in the paediatric population [5, 6].

## 2. Case Presentation

A 4-year-old boy with neurological impairment and severe growth retardation (weight 7 kg), gastroesophageal reflux and recurrent vomiting, and signs of chronic malnutrition, hypotonia, and seizure disorders was admitted for diagnostic study. Metabolic and genetic tests were unremarkable. He was treated with a venous central line to allow parenteral nutrition and electrolyte rebalancing, and he received enteral hypercaloric polymer diet per probe, which was well tolerated. To facilitate safe feeding, a PEG was positioned. The procedure was performed in general anaesthesia using a flexible gastroscope, after the stomach was insufflated; the gastric puncture was performed after transillumination. A cannula was inserted into the stomach under visual control and the assistant verified its correct positioning by air aspiration. Through the cannula sheath, a guide wire was passed, which was grasped by the endoscopist and drawn out the mouth together with the gastroscope. The thread loop of the external end of the PEG tube was fastened to the guide wire, drawn down through the oesophagus into the stomach, and out through the puncture site until the internal fixation plate had drawn the anterior wall of the stomach against the abdominal wall.

The patient was febrile the day after the procedure, but he became nonpyretic 24 hours after antibiotic therapy; blood tests and blood cultures were negative. In the following 3 weeks, the child showed signs of food intolerance such as vomiting, abdominal distension, and diarrhoea with weight stasis. The feeding formulas, administered by PEG, were modified using semielementary and elementary formulas and administered with slow infusion feeding schemas, but no improvement of diarrhoea was observed.

**Figure 1:**
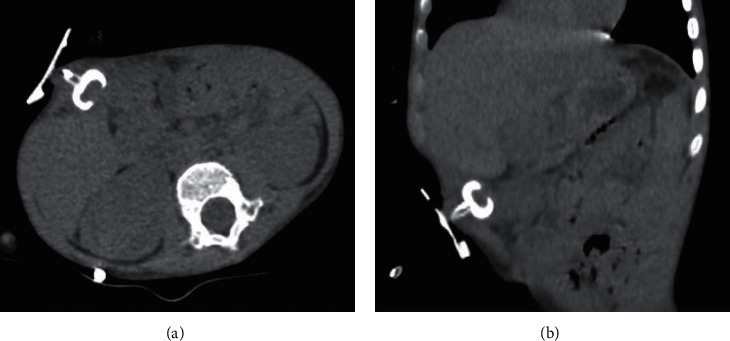
In transverse cut (a) and coronal cut (b) of CT exam, the gastrostomy tube was correctly positioned in the gastric antrum pulled to the right.

He presented twenty-two days after PEG placement, a fever spike with abdominal pain, and leakage of faecaloid material from gastrostomy. A thoracoabdominal CT scan was performed, and no signs of pneumoperitoneum and no effusion were documented. The PEG was correctly positioned in the gastric antrum pulled to the right (Figure 1).

**Figure 2:**
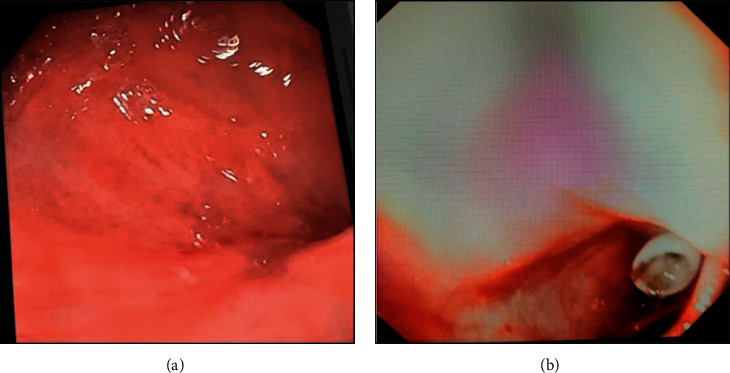
(a) Gastric view of endoscopic exam; no gastrostomy bumper was visualized in the stomach; the gastric wall was intact. (b) In colonoscopy, the gastrostomy bumper was visualized at the level of the right colonic flexure.

The secretion of faecaloid material from gastrostomy continued; therefore, the child was sedated to perform gastroscopy. No gastrostomy bumper was visualized in the stomach; the gastric wall was intact (Figure 2(a)). A colonoscopy was performed, and the gastrostomy bumper was visualized at the level of the right colonic flexure (Figure 2(b)). During the same endoscopic procedure, gastrostomy was removed.

During colonoscopy, the dislocated gastrostomy was removed, and a conservative management of colocutaneous fistula, which closed after two weeks, was adopted. Antibiotic treatment was performed, and enteral nutrition per probe with polymer diet was administered with good tolerance. About one month later, a laparoscopic-assisted gastrostomy was placed; any adhesion was found in the abdomen, except the right colonic flexure adhering to the wall.

Forty-eight hours after LAG procedure, the child received alimentation by gastrostomy with polymer formulas, which was well tolerated. He reached full enteral feeding after ten days, and he was discharged. The gastrostomy works after one year of follow-up still well, and no major complications were recorded; the patient gained weight and no further hospitalisation occurred. He was treated for a peristomal granulation with silver nitrate with good results.

## 3. Discussion

Various published studies suggest that PEG is associated with significant morbidity despite operators' experience and appropriate patients selection. Gastrocolic, gastrocolocutaneous, or colocutaneous fistula is one of the most common major complications of PEG, and the incidence is 2-3% [7]. Fistula formation is mediated by G-tube penetration of an interposed colon between the stomach and abdominal wall during the initial insertion. Various mechanisms had been proposed to explain this event, e.g., one is the inadvertent puncture of the transverse colon due to its close proximity to the stomach during PEG insertion. Transillumination used during endoscopy is unsafe because of thinner tissue thickness in children, especially if undernourished. The colic transposition is not avoidable [8]. Risk factors include adhesions from previous laparotomy, postural and spinal abnormalities, low weight, and tissue trophism [2]. It is known that better nourished children have less postoperative complications [9]. These complications can occur immediately or after months [4, 10, 11], in which children remain asymptomatic until PEG migration is completed [3]. Diarrhoea is the usual presentation. In the paediatric population, poor weight gain, yellowish diarrhoea, and stool-like discharge from PEG can be observed [4]. In our case, migration occurred immediately after the positioning procedure, but the diagnosis was complex due to confounding symptoms and misleading imaging. Conservative management of fistula may be adopted if the communication is suggestive of a colocutaneous fistula without peritoneal extravasation. The indication for an operative approach is intestinal obstruction, peritonitis, or persistent inflammation around gastrostomy [10]. The risk of PEG migration in causing gastrointestinal fistula to the colon or small bowel was also found to be more significant compared to LAG-assisted placement [8]. A laparoscopic technique confers the advantage of direct visualization of the peritoneal cavity during gastrostomy insertion instead of the blind technique used in PEG insertion. Literature demonstrates a higher major complication rate with PEG (0–11%) versus LAG (0–4%) placement [12] and placing the G-tube through a visceral structure (small bowel or colon) before entering the stomach is more common in PEG versus other approaches. In high-risk patients, laparoscopic insertion compared to PEG had less major and severe complications, and despite a longer operative time, LAG seemed to be the optimal procedure for children under 5 years [13]. Our patient underwent PEG procedure in a state of severe malnutrition in which transillumination was considered unreliable due to tissue thinness; under laparoscopic vision, colic transposition could have been avoided.

## 4. Conclusions

PEG migration and colocutaneous fistula are rare complications of PEG; they are caused by interposition of the colon between the stomach and the abdominal wall. Fever, abdominal pain, and food intolerance should raise immediate suspicion of a colonic involvement in PEG.

Clinicians should consider PEG migration in patients with refractory diarrhoea after placement of percutaneous gastrostomy. No further investigation is needed when feces material is observed at the orifice or yellowish diarrhoea occurs.

Conservative management of the colonic fistula can be achieved safely.

In patients with anatomical deformities, previous surgery, or small children (under 10 kg), a laparoscopic approach is strongly suggested.
